# Numerical Simulation of Particulate Matter 2.5 Distribution in a Roadway

**DOI:** 10.1038/s41598-018-31419-0

**Published:** 2018-09-05

**Authors:** Guorui Feng, Qi Liao, Shengyong Hu

**Affiliations:** 1 College of Mining Engineering, Taiyuan University of Technology, Taiyuan, 030024 China; 2 Green Mining Engineering Technology Research Center of Shanxi Province, Taiyuan, 030024 China

## Abstract

Large amounts of dust particles pose serious hazards to the health and safety of China’s coal miners during roadway blasting processes. It is known that among these dust particles, Particulate Matter 2.5 (PM_2.5_) does the greatest amount of harm. In order to study the distributions of the PM_2.5_ in roadway blasting processes, a mathematical model of the gas-solid two-phase flow was established in this study, which was based on a Direct Simulation Monte Carlo Method (DSMC). Then, a multiphase flow program was developed. This study’s results indicated that following the blasting processes, fine dust particles gradually floated up and were suspended for long durations in the underground roadway space. The medium-sized dust particles slowly sink to the ground and were eventually expelled before settling to the floor of the roadway. The coarse particles were rapidly settled to the roadway floor. It was determined that the PM_2.5_ particles in the front end of the dust group could not be quickly diluted, and the concentrations were high until it is expelled from the roadway, whereas the PM_2.5_ dust particles in the back end of the underground roadway were found to be gradually diluted. Eventually, the PM_2.5_ concentrations exhibited an alternating thin to dense phase distribution. When compared with the Particulate Matter 5 (PM_5_), it was found that the PM_2.5_ was more difficult to discharge, and easily formed serious PM_2.5_ dust air pollution. This study’s results were determined to be conductive to the future control of PM_2.5_ in the underground roadway blasting processes.

## Introduction

Recently, cases of pneumoconiosis in China have been increasing by 25,000 each year, and there have been more than 6,000 deaths as the result of coal dust inhalation^1^. Mining operations, such as underground roadway blasting processes, tend to generate dust in both the respirable and non-respirable size range^2^. Respirable dust is a main factor which has led to coal workers’ pneumoconiosis^3^. PM_2.5_ is classified as a respirable dust, and is known to have toxic effects on the cardiovascular system by directly entering into the human pulmonary alveolus^4^. Therefore, in order to ensure the health and safety of China’s miners, more attention should be paid to the PM_2.5_ dust particles generated in underground roadway blasting processes.

At the present time, PM_2.5_ is being widely studied in atmospheric environments all over the world^5–7^. There is no standard of PM_2.5_ concentration in the dust control of coal mines. In order to control PM_2.5_ pollution in coal mines, a ground atmospheric standard for PM_2.5_ was introduced^8–10^. Meanwhile, the PM_2.5_ in coal mines is different in concentration and composition, as well as other aspects^11^. Therefore, examining the distributions of PM_2.5_ in coal mines has become a very urgent matter.

Numerical simulation is a highly efficient method which has been widely adopted to study dust distributions in coal mines^12^. Nie *et al*.^13^ used Fluent software to simulate the total dust migration regulations of full rock excavation roadway under different airflow rates. Also, Niu *et al*.^14^ conducted a study regarding the movement behaviors of respirable dust, as well total dust distributions in mine excavation face using Fluent software. Toraño *et al*.^15^ utilized CFD software to evaluate total dust distribution regulations with two types of local ventilation methods in a fully mechanized mining face. Kurnia *et al*.^16^ conducted a study regarding the distribution of dust particles with different diameters in excavation face with different ventilation modes using CFD software. Wang *et al*.^17^ used a discrete phase model to simulate the total concentrations of dust and respirable dust in the ventilation processes of the roadway working faces in coal mine. Qin *et al*.^18^ studied the dust distributions in excavation roadways with dust removal and ventilation systems which is long pimping and short pressing, using Fluent software. Nie *et al*.^19^ applied Fluent software to study the changes in airflow fields in hard rock mechanized mining faces. Chen *et al*.^20^ applied Fluent software to simulate the dust distributions following stope blasting processes. However, the aforementioned research studies were mainly focused on the distributions of the total dust or respirable dust in the mechanized working faces of mines. To date, very little attention has been paid to the distributions of PM_2.5_ particles in underground roadways following mine blasting processes.

Therefore, in order to address this issue, a gas particle two-phase flow model was developed in this study for the examination of PM_2.5_ distributions and the resulting air pollution hazards in underground mining roadways following blasting processes.

## Method

### Gas-solid two-phase models

The discrete particle phase was studied in this study based on the Euler method. The continuous gas phase was studied based on the Lagrange method. This study also studied the gas-solid two-phase flow following blasting processes. Furthermore, based on the DSMC method, the collisions between particles were taken into consideration. Due to the relatively low concentrations of the particle phase, the coupling effects between the gas and solid phases were neglected in this study’s investigations, and only the effects of the gas phase on the solid phase were considered^21^. The momentum equation of the gas phase, along with the equation of continuity were presented as follows^22^:

1 $$\{\begin{array}{c}\frac{\partial ({u}_{j})}{\partial ({x}_{j})}=0\\ \frac{\partial ({u}_{i})}{\partial t}+\frac{\partial ({u}_{i}{u}_{j})}{\partial {x}_{j}}=\frac{1}{{\rho }_{{\rm{g}}}}(-\frac{\partial p}{\partial {x}_{i}}+\frac{\partial ({\tau }_{ji})}{\partial {x}_{j}}+{\rho }_{{\rm{g}}}{\rm{g}})\end{array}$$

where *τ*_ji_ represents the turbulent stress tensor; *u*_j_ and *u*_i_ denote the velocity; and *i*, *j = *1, 2, 3 represent the x, y, and z directions, respectively; *t* represents the unit vector in the tangential direction; *ρ*_g_ is gas phase density; g is gravitational acceleration.

2 $${\tau }_{ji}=(\mu +{\mu }_{t})(\frac{\partial {\mu }_{j}}{\partial {x}_{i}}+\frac{\partial {u}_{i}}{\partial {x}_{j}})-\frac{2}{3}{\rho }_{{\rm{g}}}\kappa {\delta }_{ij}$$

where *δ*_ij_ represents the Kronecker constant; *κ* represents the turbulent kinetic energy; *μ*_t_ denotes the turbulent viscosity; and *μ* denotes the dynamic viscosity.

The forces on the particle phase were mainly considered, including the forces of the particle-particle interactions; forces of the particle-wall interactions; and forces of the particles and airflow^23,24^. There were multiple forces of particle-gas interactions present, including the Basset, Magnus, Saffman, pressure gradient, false quality, and drag forces. However, the gravity force was only considered among the field forces. Also, only the drag force was taken into consideration among the fluid forces. The related equations were as follows:

3 $${f}_{d}=\beta (v-u)$$

4 $${u}_{r}=u-v$$

where *f*_d_ represents the drag force; *v* is the solid phase velocity; and *u* is the gas velocity.

5 $${\rm{Re}}={D}_{P}|{u}_{r}|/v$$

6 $${\rm{\beta }}=\frac{3}{4}{C}_{D}\frac{|v-u|\rho (1-\varepsilon )}{{D}_{p}}{\varepsilon }^{-2.7}$$

where Re is Reynolds number; *D*_p_ represents the particle diameter; *ε* is the voltage; and *ρ* is the gas phase density.

7 $${C}_{D}=\{\begin{array}{c}24(1+0.15R{e}^{0.687})/\\ \,0.43\,\end{array}\begin{array}{c}Re\,Re\le 1000\\ Re > 1000\,\,\,\end{array}$$

Then, the trajectories of the sample particles were studied based on the DSMC model. Due to the effects of the particle rotations, the particle-particle collisions were determined using probability. The related equations were as follows:

8 $${m}_{1}({V}_{1}-{V}_{1}^{(0)})=J$$

9 $${m}_{2}({V}_{2}-{V}_{2}^{(0)})=-\,J$$

where *m* is the mass; *J* is the impulse exerted on Particle 1, acts on Particle 2 as the reaction; *V* represents the velocity. In the following two equations, the subscripts 1 and 2 represent the two particles. Also, the superscript (0) represents the values before collision.

10 $${I}_{1}({\omega }_{1}-{\omega }_{1}^{(0)})={r}_{1}n\times J$$

11 $${I}_{2}({\omega }_{2}-{\omega }_{2}^{(0)})={r}_{2}n\times J$$

where *I* represents rotational inertia of particle; *r* stands for the particle radius; and *n* stands for the normal unit vector directed from Particle 1 to Particle 2 at the moment of contact.

12 $$\frac{n\cdot {G}^{(0)}}{|{G}_{ct}^{(0)}|} > \frac{2}{7}\frac{1}{f(1+e)}$$

13 $${V}_{1}={V}_{1}^{(0)}-[(1+e)(n\cdot {G}^{(0)})n+\frac{2}{7}|{G}_{ct}^{(0)}|t]\frac{{m}_{2}}{{m}_{1}+{m}_{2}}$$

14 $${V}_{2}={V}_{2}^{(0)}+[(1+e)(n\cdot {G}^{(0)})n+\frac{2}{7}|{G}_{ct}^{(0)}|t]\frac{{m}_{1}}{{m}_{1}+{m}_{2}}$$

15 $${\omega }_{1}={\omega }_{1}^{(0)}-\frac{5}{7{r}_{1}}|{G}_{ct}^{(0)}|(n\times t)\frac{{m}_{2}}{{m}_{1}+{m}_{2}}$$

16 $${\omega }_{2}={\omega }_{2}^{(0)}-\frac{5}{7{r}_{2}}|{G}_{ct}^{(0)}|(n\times t)\frac{{m}_{1}}{{m}_{1}+{m}_{2}}$$

17 $$\frac{n\cdot {G}^{(0)}}{|{G}_{ct}^{(0)}|} < \frac{2}{7}\frac{1}{f(1+e)}$$

18 $${V}_{1}={V}_{1}^{(0)}-(n-ft)(n\cdot {G}^{(0)})(1+e)\frac{{m}_{2}}{{m}_{1}+{m}_{2}}$$

19 $${V}_{2}={V}_{2}^{(0)}+(n-ft)(n\cdot {G}^{(0)})(1+e)\frac{{m}_{1}}{{m}_{1}+{m}_{2}}$$

20 $${\omega }_{1}={\omega }_{1}^{(0)}+\frac{5}{2{r}_{1}}(n\cdot {G}^{(0)})(n\times t)f(1+e)\frac{{m}_{2}}{{m}_{1}+{m}_{2}}$$

21 $${\omega }_{2}={\omega }_{2}^{(0)}+\frac{5}{2{r}_{2}}(n\cdot {G}^{(0)})(n\times t)f(1+e)\frac{{m}_{1}}{{m}_{1}+{m}_{2}}$$

where *f* refers to the friction coefficient of the Coulomb Friction Law; and *e* refers to the coefficient of the restitution.

22 $${G}^{(0)}={V}_{1}^{(0)}-{V}_{2}^{(0)}$$

23 $${G}_{ct}^{(0)}={G}^{(0)}-({G}^{(0)}\cdot n)n+{r}_{1}{\omega }_{1}^{(0)}\times n+{r}_{2}{\omega }_{2}^{(0)}\times n$$

24 $${\rm{t}}=\frac{{G}_{ct}^{(0)}}{|{G}_{ct}^{(0)}|}$$

where *G*^(0)^ is the relative velocity of the particle centers before collision occurs; *G*_ct_^(0)^ denotes the tangential component of the *G*^(0)^ of the contact point prior to collision occurring.

In this study, the roadway wall was regarded as an infinite sphere. A particle-to-particle collision model was established to deal with the collisions between the particles and roadway wall. Then, the corresponding program development of the particle movements was independently completed.

### Geometrical model

**Figure 1:**
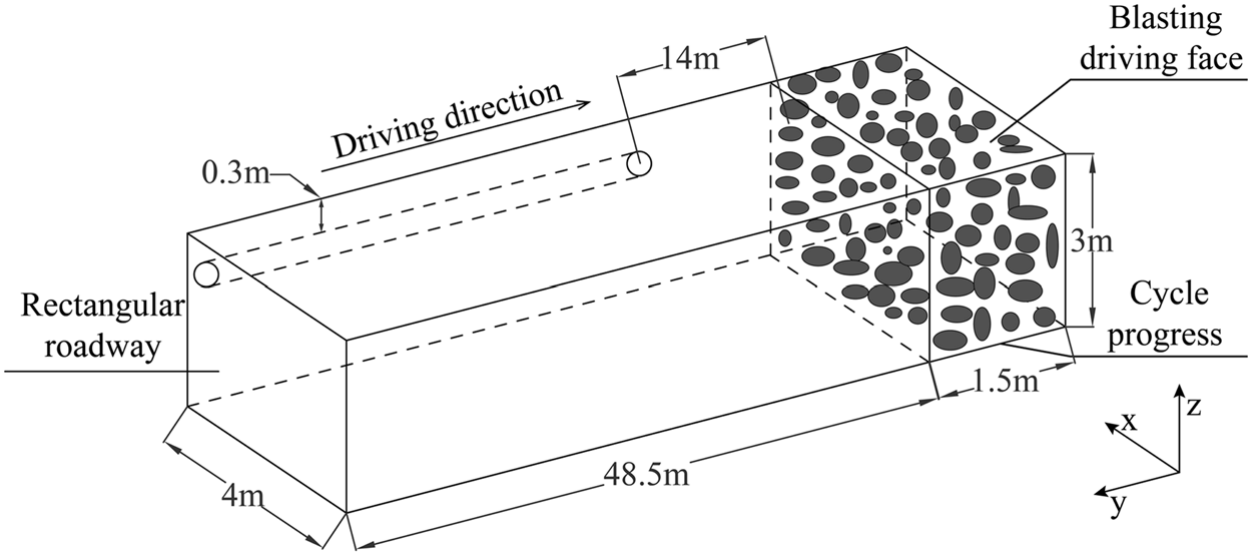
Geometric model of the blast driven roadway.

The Wulan Coal Mine is located in the Ningxia Hui Autonomous Region of China. Large amounts of coal dust are produced during the working face blasting processes in the Wulan Coal Mine due to its soft coal seam. These coal dust particles float in the air with an average concentration of 1,500 mg/m^3^. A working face in China’s Wulan Coal Mine was selected as a physical prototype, as shown in Fig. 1. The study area’s underground rectangular roadway was 4 m in width, and had a height of 3 m. The ventilation duct which was located in the underground roadway had a 0.8 m diameter. The distance from the outlet of the ventilation duct to the working face was 14 m, and was 0.3 m from the roof. The airflow velocity in the outlet of ventilation duct was 10 m/s. The coal dust spray following the blasting had a speed of 6 m/s. Table 1 lists the simulation parameters of this study’s geometric model.

**Table 1:** Calculation model parameters.

Simulation parameters	Value
Air viscosity (m^2^/s)	0.000017894
Air density (kg/m^3^)	1.225
Dust density (kg/m^3^)	1500
Time step for particle (s)	0.000025
Simulated particles flow time (s)	180
Initial dust concentration (mg/m^3^)	1500
Particle median diameter	0.000055
Particle dispersion index	2.95
Maximum particle diameter (μm)	100
Minimum particle diameter (μm)	0.1
Gas phase grid number	530000
Coefficient of restitution between particles	0.8
Coefficient of restitution between particle and wall	0.6
Friction coefficient between particles	0.3
Friction coefficient between particle and roadway wall	0.4

## Results and Discussion

### Airflow fields

**Figure 2:**
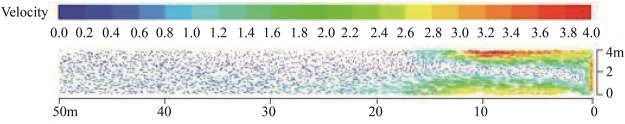
Velocity distributions of airflow fields in the breathing zone.

In order to study the airflow fields in the study area after the blasting processes, the ordinate Z = 1.5 was taken as the height of the breathing zone. Figure 2 shows the velocity distributions in the breathing zone following the blasting processes.

As shown in Fig. 2, within the front-end of the roadway, the airflow velocity was approximately 3.2 m/s. Its maximum value on the left-hand side of the roadway was 4 m/s, while it was 2.8 m/s on the right. Near the left wall of the front-end, the direction of the blasting shock wave was the opposite of the airflow. Therefore, the airflow velocity near the left wall of the front-end was significantly lower than that in the outlet of the ventilation duct. Due to the viscous effects, the airflow velocities around the blast working face and close to the right wall were obviously lower than those on the left-hand side.

In the roadway areas ranging from 0 to 25 m away from the driving face, an obvious vortex region with a banded vortex center was obvious. The airflow velocity in the vortex center was approximately 0.4 m/s, which was significantly lower than that close to the blast driven face and near both sides of the underground roadway. In the regions located more than 25 m from the driven face of the coal mine roadway, the airflow velocity gradually became weakened to below 1.0 m/s. This was found to be due to the friction between the roadway wall and airflow, and the viscous force of the airflow itself. It is the leading role of airflow return that the airflow field became totally stabilized in.

### Particle trajectories

**Figure 3:**
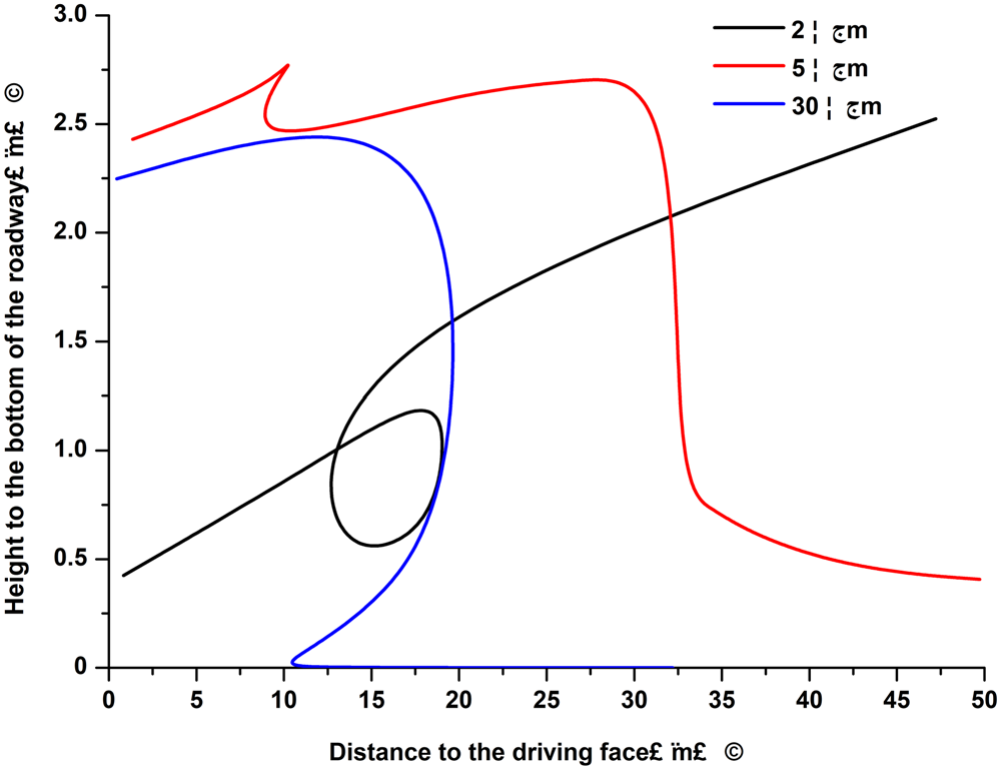
Contrasts in the trajectories of the different particle sizes.

In this study, dust particles with diameters of 2 μm, 5 μm, and 30 μm were taken as examples. Figure 3 shows the particle movement trajectories over time following the blasting processes.

As shown in Fig. 3, dust particles with different sized diameters were suspended in the air in the roadway from 0 to 10 m away from the working face. It was found that the blasting shock waves leading roles in the initial spraying velocities of the dust particles. In the areas located more than 10 m away from the working face, the wind-drag forces gradually played leading roles, rather than the blasting shock waves. The particles displayed obvious discrepancies in their movement trajectories due to the different gravity forces. The 2 μm particles were too light to settle down naturally. Therefore, they moved upwards and continue floating due to the drag forces. The middle-sized dust particles (5 μm) exhibited gradual sinking movement trajectories due to moderate gravity forces. Under the effects of the airflow, these particles moved forward along the upper position of the floor of roadway until being discharged^25,26^. The coarse particles (30 μm) settled rapidly to the floor due to the leading effects of sufficient gravity forces. The coarse particles then slid forward along the floor due to the wind-drag forces in the roadway. Eventually, due to friction forces, the coarse particle migration ceased to form dust retention.

### PM_2.5_ distributions

**Figure 4:**
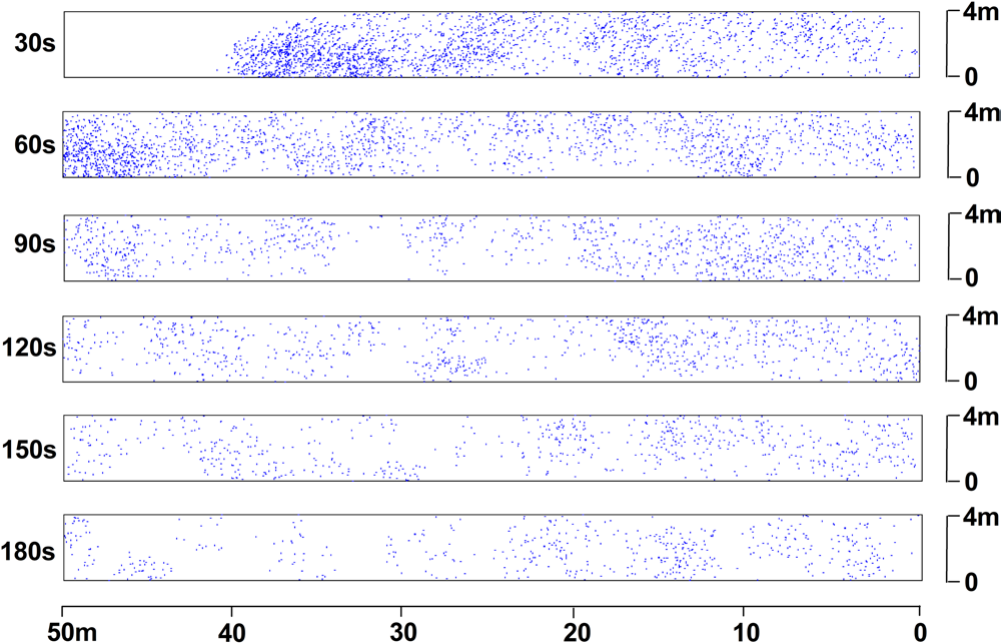
PM_2.5_ particle distributions within the breathing zone at different times.

**Figure 5:**
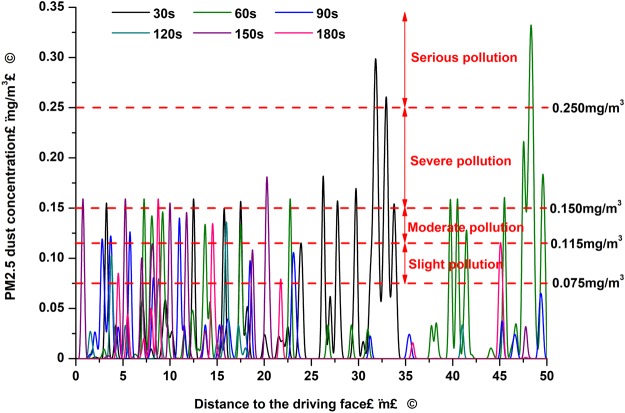
PM_2.5_ concentrations along the roadway at the height of 1.5 m in the breathing zone.

This research study observed the PM_2.5_ distributions following the blasting processes at each time point, which were at 30, 60, 90, 120, 150, and 180 seconds, respectively. Figures 4 and 5 detail the PM_2.5_ distributions at a breathing height of 1.5 m, and in the region of the 0.25 m upper and lower sections of the breathing height, respectively.

As shown in Fig. 4, within 30 seconds of the blasting, due to the strong blasting shock waves, large amounts of dust particle spray with high velocity were detected in the roadway space. During that time, high concentrations of dust particles in the PM_2.5_ group passed across the blast working face. These dust particles posed serious threats to workers in the active working face. The dust particles in the front end of the dust group were not timely and were effectively diluted. This concentration maintained a high level until being discharged. Meanwhile, the dust particles in the back end of the roadway were gradually diluted. These lower concentrations displayed stable distributions. This was mainly due to the higher airflow velocity in the front end of roadway, as shown in Fig. 2. As time passed, the dust particles in the high concentration areas were completely discharged from the roadway outlet as airflow. Eventually, only the PM_2.5_ of lower concentration levels were suspended in the roadway space. Overall, the PM_2.5_ represented alternately thin to dense distributions.

As shown in Fig. 5, the peak value of the PM_2.5_ concentration was 0.33 mg/m^3^ in the area 31.75 m away from the working face, at t = 30 seconds after blasting occurred. It was found to be 0.36 mg/m^3^ at 48.25 m away from the working face, at t = 60 seconds. These findings presented serious pollution levels according to China’s atmospheric quality standards. As time passed, the PM_2.5_ accumulation zone with high concentrations gradually moved forward, and was fundamentally expelled from the roadway after approximately 60 seconds. At that point, the PM_2.5_ concentrations started to slowly decline, and stabilized below 0.24 mg/m^3^. In the region located 0 to 25 m away from the working face, the PM_2.5_ concentration remained at a high level. The fine dust was difficult to discharge as the airflow moved due to the vortex region located in that section, as shown in Fig. 2. At t = 180 seconds, the PM_2.5_ concentrations located in parts of the roadway zones were still more than 0.075 mg/m^3^, and even more than 0.15 mg/m^3^, presenting moderate pollution levels.

### Comparison of the dust concentration levels

**Figure 6:**
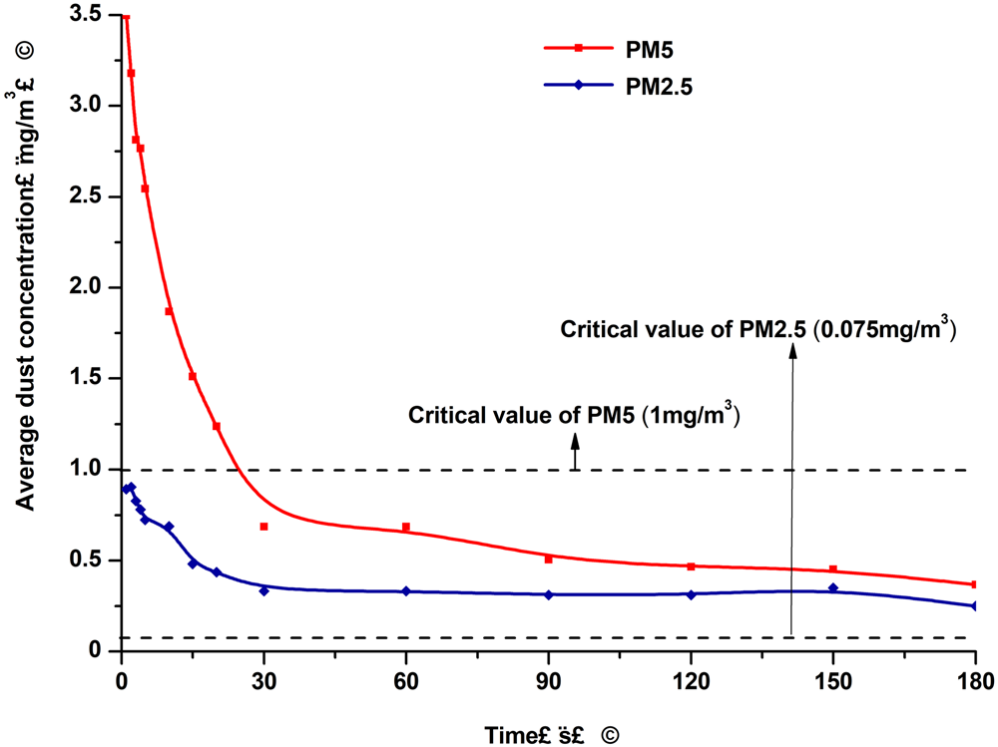
Comparison of the dust particle concentrations of the PM_2.5_ and PM_5_.

Fig. 6 shows the statistical results of the average concentrations of PM_2.5_ and PM_5_ in the three-dimensional roadway following the blasting processes. As shown in Fig. 6, the PM_5_ concentration after blasting was 3.5 mg/m^3^, which was approximately three to four times that of the PM_2.5_. At 0 to 30 seconds after the blasting, the PM_5_ and PM_2.5_ concentrations had rapidly decreased. The decreased velocities of the PM_5_ were obviously higher than that of the PM_2.5_. After 30 seconds, the PM_5_ concentration decreased relatively slowly, and maintained a downward trend. Meanwhile, the PM_2.5_ concentrations had only weakly declined or faintly undulated. As time continued to pass, the PM_2.5_ and PM_5_ concentrations both exhibited almost unchanged or extremely slow declining trends. Finally, the PM_5_ concentrations stabilized at 0.5 mg/m^3^, which was below its critical value of 1 mg/m^3^. During the same period of time, the PM_2.5_ concentrations stabilized at 0.3 mg/m^3^, which was well over its critical value of 0.075 mg/ m^3^.

### Verification of the numerical results

**Figure 7:**
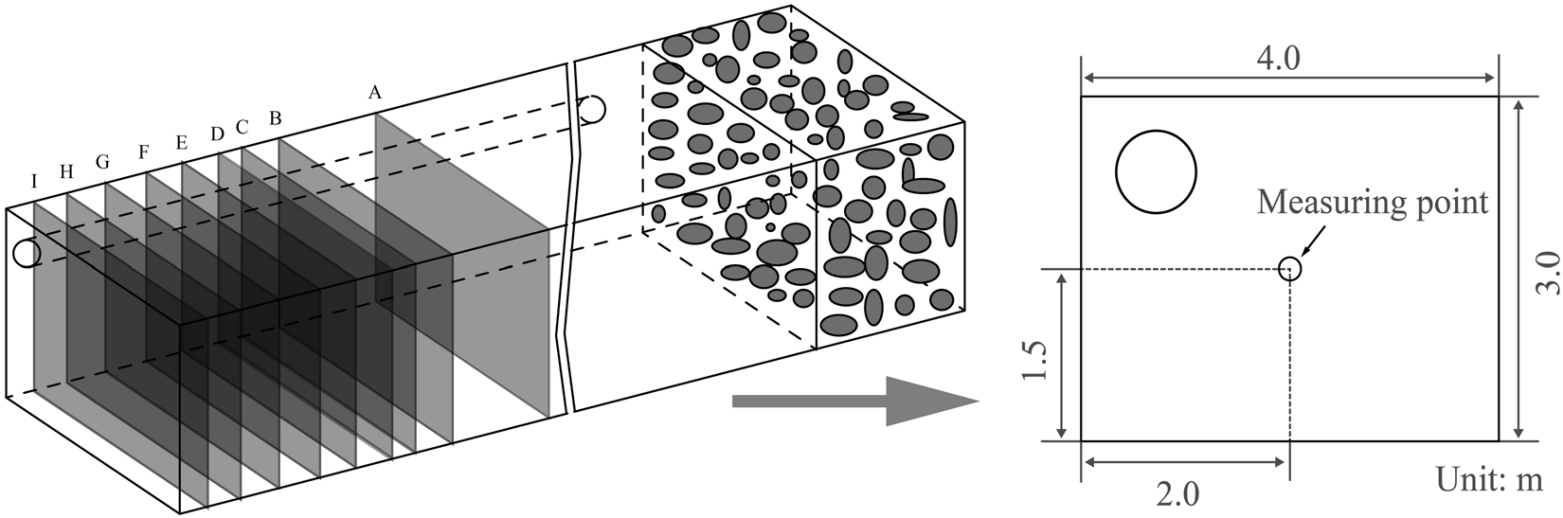
Diagram of the measurement point arrangement.

In order to verify the accuracy of this study’s numerical results, the field data of a blast driven working face of the Wulan Coal Mine were measured. In accordance with the tunnel size and the arrangement of the production site, nine measuring points were set at nine cross-sections A, B, C, D, E, F, G, H, I with distances from the working face, at 30, 35, 37, 38, 40, 42, 44, 46, and 48 m, respectively, as shown in Fig. 7. All of the measuring points were fixed at heights of 1.5 m above the roadway floor, and at the same distances from the both sides of the roadway. It was difficult to precisely measure the lower PM_2.5_ concentrations using a type of intrinsically safe extant dust detecting device. Also, the different sized dust particles exhibited similar dust concentration change tendencies^26,27^. Therefore, the dust concentration data of all the dust in the working face of the Wulan Coal Mine were collected by the intrinsically safe extant dust detecting type device in this study. The data were measured within 180 seconds after the blasting processes occurred. A mine dust sampler (AKFC-92A, Zhengzhou Huazhi Electronic Technology Co., Ltd., China), was used in this study for the collection of the dust particles.

**Figure 8:**
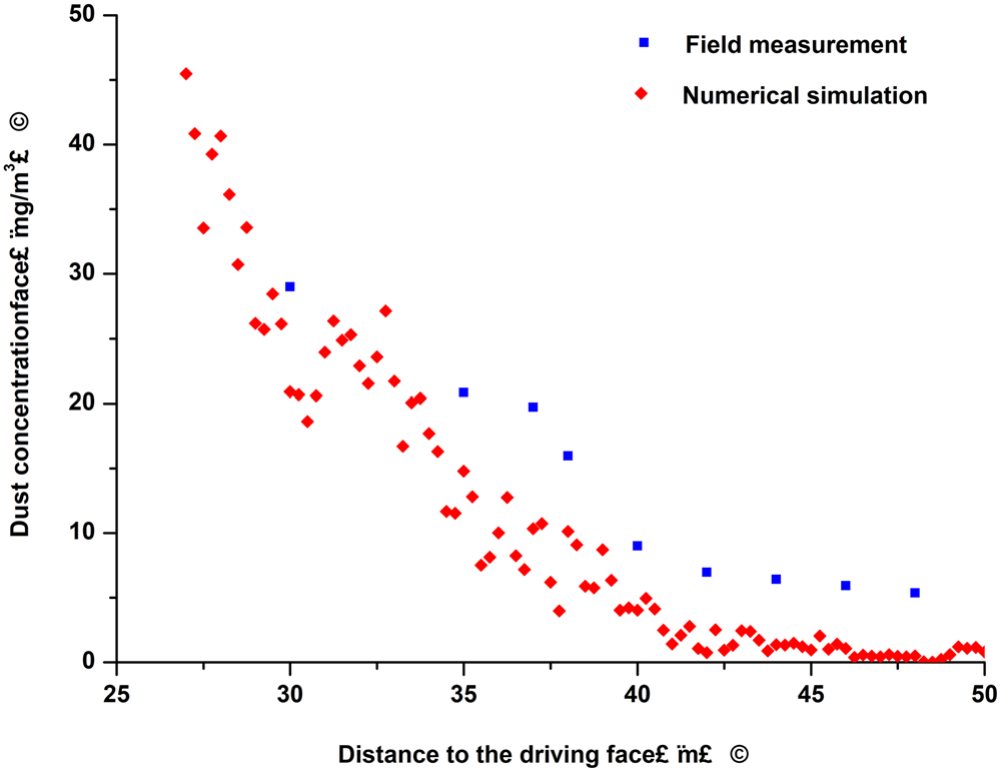
Comparison of the numeral and measured results.

This study’s comparison with the simulation results is illustrated in Fig. 8. As can be seen in the figure, the simulation results were found to be in good agreement with experimental data, which indicated an overall decreasing trend of the PM_2.5_ concentrations along the roadway. Due to the influences of the personnel, devices, environment, and other factors, some deviations between the two results were found to exist. In fact, the PM_2.5_ concentrations displayed a thick or dense alternation. This was determined to be mainly due to the smaller particle sizes and lighter masses of the PM_2.5_ particles, as well as the easy movement under the action of the turbulent wind-drag forces. This study’s mathematical model was determined to be accurate, and credible numerical results were obtained.

## Conclusions

In this study, the following conclusions were obtained and summarized based on above results and discussion:

1. An obvious vortex region with a banded vortex center was found to exist in the front area of the underground roadway. The airflow velocity around the working face and close to the walls was significantly higher than that in the vortex center. The airflow field became gradually weakened along the roadway, and eventually stabilized.
2. The coarse dust particles rapidly realized natural sedimentation following the blasting processes. The medium-sized dust particles moved forward along the upper position of the floor, and were discharged before settling at the floor. The fine dust particles continue floating in the air of the roadway for a long period of time.
3. Following the blasting processes, the high-concentration PM_2.5_ areas located in the front end of the dust group were not quickly diluted. However, the PM_2.5_ particles at the back end of the underground roadway had been gradually diluted. These particles exhibited an overall alternating thin to dense phase distribution. When compared with the PM_5_, the PM_2.5_ was found to be more difficult to discharge, which easily resulted in serious air pollution levels.

This study’s simulation results offer a scientific basis for the formulation of a future concentration standard for PM_2.5_ dust particles in China’s underground coal mine operations.
